# Making an Aluminum Plate

**Published:** 1906-02-15

**Authors:** Geo. D. Sitherwood


### MAKING AN ALUMINUM PLATE.

BY GEO. D. SITHERWOOD.

Pure aluminum, so-called, the best that is manufactured to-day, is softer and not so durable as that made fifteen or twenty years ago. For a swaged plate take nothing of a lighter gauge than No. 20 Brown & Sharpe's Standard. Use the ordinary zinc die and lead counter. See that your plate crosses the die from side to side with the grain of the plate in rolling. Keep tissue paper between the plate and the die and also the counter, you will thus prevent particles of lead and zinc from being driven into the plate. Anneal three or four times, being careful to not overheat. For the last drive in swaging use no paper. See that the edge of the plate is high enough for turning a rim at least from one-eighth to three-sixteenths of an inch in width, by trying in the mouth.

With a flat nosed pair of pliers begin at the heel of the plate and bend as much of the plate outward as desired for a rim. Go around the plate once, bending it out a little, anneal slightly, then go around once or twice more, bending it down a little each time. Then place the plate on the die, and with a piece of hard wood and hammer drive the rim well down, and finally, put it in the lead counter die and drive it firmly home.

Wax is then placed around the edge of plate and the bite taken in the usual manner. The plate is put on the articulator and teeth set on and waxed up. On removal from the articulator, if possible, try in the mouth first, then flask the same as for a rubber plate. Separate, remove wax and put in the rubber, a good quality of pink is the best, using a piece of the cloth which comes with rubber placed over the aluminum plate. When the flask is pressed together in boiling water, remove and separate, take out any surplus rubber or if not perfectly filled add some more. Now carefully remove the plate, place on the zinc die, and with a small narrow-bladed chisel proceed to lift the rim by having an assistant to strike the chisel with a hammer; hand pressure will not do as it will invariably warp the plate. When the rim is driven out enough to form an attachment for the rubber, then begin to etch on the palatal surface where a line has been scratched with a sharp instrument at the very edge of the wax attachment of the teeth. Set the small chisel first toward the inside of the line with the handle turned outward and let your assistant strike a blow sufficiently hard to make a distinct cut but not through the plate. Make each cut far enough apart to permit of a cut being made between each, by turning the chisel in the opposite direction of a return row. For the remainder of the etching use any sharp chisel corner, going around the plate between this palatal row and the outer rim, making alternate rows by turning the chisel in the opposite direction each time. Apply a little olive oil and turpentine to plate and the chisel will cut more readily. A direct easy blow with the hammer by an assistant while the plate is firmly held on the die is the only way to successfully etch an aluminum plate.

A pressed aluminum plate without a turned rim and a perfectly fitting metal die to etch it on, is comparatively worthless, and will be a great disappointment to any one attempting that process.

After the plate is etched thoroughly cleanse it with sulphuric acid and boiling water, and afterward the soda bath. Wash and dry it, and varnish with sandaiac varnish all that portion of the plate where no rubber is desired, especially inside. Now place the plate again in the flask, close and vulcanize at least an hour and fifteen minutes, 320 degrees F. The plate is finished and polished almost the same as the rubber plate. A little alcohol will remove the sandarac vainish which prevented the sulphur from tarnishing the clean aluminum.

The turned rim around the edge of an aluminum plate prevents the plate from warping or changing its shape just enough to spoil the fit. It also forms a complete arrangement for holding the upper part of the rubber attachment in place.

If sufficient attention and care is given to all the details here mentioned, and such cleanliness observed as each step absolutely requires, an artificial denture will be secured, that for comfort, appearance and utility is surpassed by no other plate, unless a platinum with the porcelain finish, known as a continuous gum work. This statement is made after an experience of twenty-five years in working aluminum —*Brief.*

